# Biallelic WDR19 Variants: Systematic Analysis of Genotype–Phenotype Correlations

**DOI:** 10.1155/humu/9449547

**Published:** 2026-09-17

**Authors:** Zoe Webster, Lisa Woods, Robert Dalziel, Kylie Drake, Guillem de Valles-Ibáñez, Gemma Poke

**Affiliations:** ^1^ Genetic Health Services, Wellington Hospital, Health New Zealand Te Whatu Ora, Wellington, New Zealand, thewellingtonhospital.com; ^2^ School of Mathematics and Statistics, Victoria University of Wellington, Wellington, New Zealand, victoria.ac.nz; ^3^ Canterbury Health Laboratories, Health New Zealand Te Whatu Ora, Christchurch, Waitaha Canterbury, New Zealand; ^4^ Do IT Now, Wellington, New Zealand; ^5^ Department of Medicine, University of Otago, Wellington, New Zealand, otago.ac.nz

## Abstract

Ciliopathies represent a diverse group of inherited disorders resulting from dysfunctional cilia. A rare subset is associated with biallelic variants in *WDR19*, with fewer than 100 affected individuals reported worldwide. This study is aimed at reviewing the clinical and molecular spectrum of *WDR19*‐associated ciliopathies, with a particular focus on genotype–phenotype correlations. We report a 32‐year‐old woman with renal, ocular and hepatic manifestations attributed to compound heterozygous variants in *WDR19*, and reviewed available data from 93 previously published cases. Missense, truncating, splice‐site and copy number variants have all been reported, with renal, ocular, skeletal and hepatic involvement most frequently observed. Genotype–phenotype analyses indicated that the presence of a truncating variant was associated with increased likelihood of skeletal involvement and reduced likelihood of hepatobiliary disease compared with individuals harbouring only missense variants. Amongst individuals with only missense variants, variants located outside repeat protein domains were associated with early symptom onset (before 16 years of age) and with ocular manifestations. Notably, recurrent variants demonstrated considerable phenotypic variability. This study represents the largest review of *WDR19* variants to date and contributes to current understanding of *WDR19*‐related disease.

## 1. Introduction

Ciliopathies are clinically and genetically heterogeneous disorders caused by dysfunction of the primary cilia; antenna‐like organelles extending from the cell body [1, 2]. Autosomal recessive mutations in the genes encoding cilia‐associated proteins can result in complex multisystemic presentations, most commonly affecting the kidneys, retina, nervous system and skeleton [3, 4].

Ciliary structure and function involve bidirectional protein transport from ciliary base to tip known as the intraflagellar transport (IFT) system. *WDR19* is one of several genes implicated in intraflagellar ciliary protein function, and encodes the IFT subunit IFT144. The *WDR19* gene is located on chromosome 4p15–4p11, contains 36 exons and spans 110 kb [5]. Protein structure includes six WD repeats, three transmembrane domains and a clathrin heavy‐chain repeat [6].

In 2011, Bredrup et al. identified compound heterozygous *WDR19* mutations in a Norwegian family with cranioectodermal dysplasia/Sensenbrenner syndrome (OMIM #218330) and in a Moroccan family with isolated nephronophthisis (OMIM #614377), as well as a homozygous missense *WDR19* mutation in a Dutch family with Jeune syndrome (OMIM #208500) [1]. Over 90 individuals with *WDR19* variants have subsequently been published in the literature, with phenotypes ranging from isolated retinal dystrophy to spermatogenic failure, Caroli disease, Mainzer‐Saldino and Senior–Løken syndrome (SLS) [7–10]. Nephronophthisis associated with *WDR19* variants has been designated NPHP‐13 (OMIM #614377).

The rarity and variability of WDR19‐related disorders contribute to challenges interpreting genotype‐phenotype correlations, which to date remain poorly understood. We report a 32‐year‐old New Zealand European woman with compound heterozygous missense variants in *WDR19*, presenting with young‐onset end stage renal disease initially diagnosed as IgA nephropathy, dilated intrahepatic bile ducts consistent with Caroli disease and widespread retinal drusen. We then reviewed the available clinical and molecular data from 93 published cases of WDR19‐related ciliopathies, with the aim of exploring genotype–phenotype correlations in this rare autosomal recessive condition. This article is aimed at detailing our current understanding of WDR19 ciliopathies, with a view to providing prognostic information for newly diagnosed individuals.

## 2. Case Report

A 32‐year‐old woman presented to nephrology clinic at age 19 following incidental detection of renal impairment with an estimated glomerular filtration rate (eGFR) of 49 mL/min/1.73m^2^. She had no relevant medical history in childhood nor prior hospitalisations. Heel‐prick testing as a neonate was positive for cystic fibrosis; however, subsequent sweat testing was negative. Family history included a brother with end‐stage renal disease, who had non‐specific glomerulosclerosis on kidney biopsy at age 14 years and underwent renal transplantation at age 19.

Height was 173 cm, weight 54 kg and blood pressure 130/60 mmHg. No abnormalities were detected on physical examination. Urinalysis showed trace protein with no haematuria. Ultrasound revealed normal sized kidneys with a 1.1 cm simple cyst in the lower pole of the right kidney. Renal function deteriorated gradually with ongoing bland urinalysis. Biopsy at age 21 showed global sclerosis in 3/12 glomeruli, with mild increase in mesangial cellularity in the remainder and 40%–50% tubulointerstitial atrophy. Immunofluorescence showed patchy glomerulomesangial IgA positivity and electron microscopy showed foot process fusion with occasional electron‐dense deposits in the mesangium. She was initially diagnosed with suspected IgA nephropathy with a possible superadded tubulointerstitial nephritis.

Over the next 5 years, renal function steadily declined, with an eGFR of 13 mL/min/1.73m^2^, creatinine 398 mmol/L and blood pressure 190/86 mmHg prior to commencing peritoneal dialysis at age 26. Eight months later she received a renal transplant from her paternal aunt with immediate graft function.

Posttransplant CT abdomen was arranged in the context of painless haematuria and right iliac fossa pain. No renal calculi or cause for discomfort was found; however, dilated intrahepatic bile ducts were identified. Serial 3–6 monthly serum liver function tests were normal. Liver autoantibodies, ANA and ANCA were negative. Magnetic resonance cholangiopancreatography showed beaded dilatation of 1st and 2nd degree hepatic ducts with irregular contour and ‘central dot sign’ suggesting Caroli disease. There was no portal vein thrombosis or evidence of portal hypertension on ultrasound with Dopplers. Liver Fibroscan measured 2.0–4.0 kPa. Upper GI endoscopy with biopsies was normal with no evidence of Coeliac disease.

**Figure 1:**
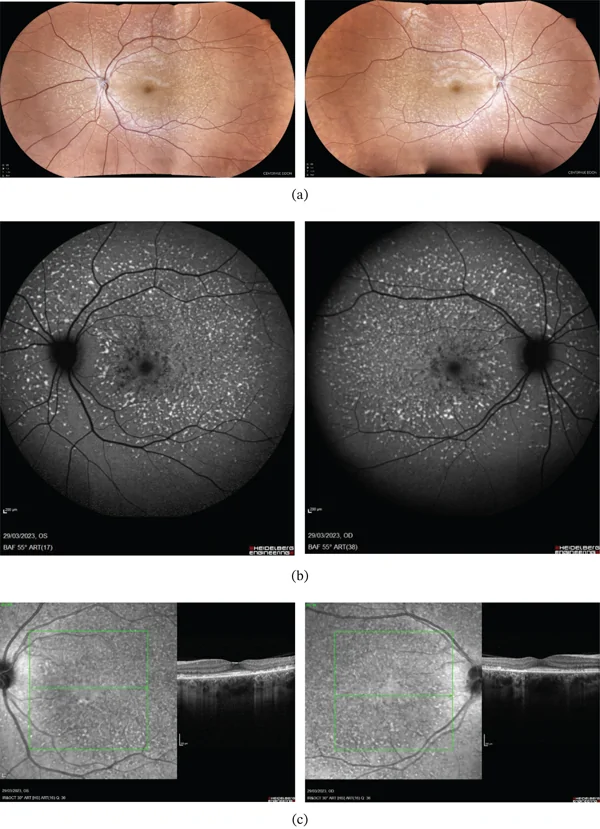
Ocular imaging from our patient. (a) Widefield retinal imaging and (b) blue‐light fundus autofluorescence showing ‘starry‐sky’ appearance. (c) Optical coherence tomography of the macular showing bilateral extensive fovea‐sparing drusen and parafoveal ellipsoid zone loss.

Routine optometry review at age 30 identified chronically poor night vision and widespread drusen throughout the retina. Ophthalmology review confirmed bilateral extensive fovea‐sparing drusen and parafoveal ellipsoid zone loss, with a ‘starry sky’ appearance on fundus autofluorescence (Figure 1).

The ocular findings in combination with her renal history prompted genetic testing at age 31, performed using next‐generation sequencing after written informed consent. One hundred seventy‐four genes associated with unexplained young‐onset end‐stage renal disease (PanelApp UK v3.0) were sequenced in addition to the mitochondrial genome. Compound heterozygous missense variants in *WDR19* were identified; a maternally inherited NM_025132.4:c.817A > G (p.Asn273Asp) and paternally inherited c.3515G > A (p.Gly1172Glu). According to ACMG/AMP guidelines, c.817A > G was classified as likely pathogenic (PM2 + BP4_Mod + PM3_VeryStrong) [11]. The paternally inherited c.3515G > A was classified as a variant of uncertain significance (PM2 + BP4_Supp + PM3). Segregation of the variants and further phenotypic assessment in her affected sibling could not be fulfilled.

## 3. Materials and Methods

### 3.1. Genetic Analysis

Genetic testing was performed by Blueprint Genetics using whole‐exome sequencing (WES), with analysis restricted to a virtual nephronophthisis‐associated gene panel (PanelApp UK unexplained young onset end‐stage renal disease v3.0; 174 green (high evidence) genes; March 2023). Genomic DNA was fragmented and sequencing libraries were prepared by adapter ligation, size selection and PCR amplification. Exonic and selected intronic regions were enriched using hybridisation‐based target capture, followed by paired‐end sequencing (2 × 150 bp) using Illumina sequencing‐by‐synthesis technology.

Sequence reads were aligned to the GRCh37/hg19 human reference genome using BWA‐MEM. Variant calling was performed using GATK‐based algorithms (Sentieon), with annotation incorporating population and clinical databases including gnomAD, ClinVar and HGMD. Copy‐number variants, including exon‐level deletions and duplications, were assessed from sequencing‐depth data using Blueprint Genetics′ validated bioinformatic pipeline.

Variants were interpreted in the context of the clinical phenotype and classified using Blueprint Genetics′ variant classification framework, based on the ACMG/AMP guidelines [11].

Of the 174 green genes included in PanelApp UK unexplained young onset end‐stage renal disease v3.0, six genes/regions (*CFHR1*, *COQ8B*, *ITSN1*, *PODXL*, *TNS2* and *TXNDC15*) were not included in the virtual panel analysis. Coverage limitations also excluded individual exons within four genes: *CC2D2A* exon 7, *KIAA0586* exons 6 and 33, *RPGRIP1L* exon 23 and *TCTN1* exons 2 and 6.

Since the original analysis was performed, the PanelApp UK panel has expanded. The current unexplained young onset end‐stage renal disease panel (v13.3, May 2026) comprises 256 green genes, reflecting the continuing evolution of the genetic evidence base for young‐onset renal disease. Regarding ciliopathy genes specifically, 123 genes associated with primary ciliopathies were not covered by the panel performed; four of these (*GLIS2, ZNF423, DCDC2* and *ADAMTS9*) are associated with nephronophthisis [12].

### 3.2. Selection and Identification of *WDR19* Variants

Previously reported cases of WDR19‐related disease were identified through a systematic search of databases PubMed and Embase. Search strategy used the keyword ‘WDR19’ and included articles published since the database inception until November 2025, limited to the English language and those on human populations.

Clinical data was recorded according to human phenotype ontology (HPO) terms, and subsequently grouped into organ‐specific categories (Table S1). Age was recorded as either paediatric (< 16 years) or adult‐onset for the first clinical manifestation identified. Patients were cross‐checked across publications to avoid duplication.

Variants were categorised based on their predicted molecular consequence, for example, truncating, missense, splicing, exon deletion or duplication. The location of missense variants in relation to conserved protein domain regions was identified using the UniProt website [13].

To ensure robustness of genotype–phenotype statistical analysis, cases were excluded if individual clinical data was unavailable, or if they harboured a single heterozygous *WDR19* mutation only, or if they harboured only variants of uncertain significance (VUS).

Reporting and curation of variant classification differed between articles. The Clinvar database and genomic analysis platforms VarSome and Franklin were used to screen for discordant interpretations on pathogenicity [14–16]. Variants classified as either likely pathogenic or pathogenic (LP/P) were included in further analysis. Where individuals were homozygous or compound heterozygous for VUSs only, those variants were independently recurated according to ACMG criteria. Remaining VUSs were included in inferential analysis only if inherited in trans with a LP/P variant.

### 3.3. Statistical Analysis (Subanalysis)

Statistical analysis was performed using R Version 4.5.1 and RStudio software [17, 18]. Descriptive statistics (counts and percentages) were used to summarise baseline patient characteristics (variant combination, domain location, age at first presentation, phenotypic category). Associations between variant combinations and clinical variables were assessed using Pearson′s chi‐squared test, with Fisher′s exact test used when expected counts were small. Logistic regression was used to evaluate the effects of variant combination and domain location on the likelihood of exhibiting each phenotype. When complete or quasi‐complete separation was present, Firth′s bias‐reduced logistic regression was applied. A *p* value < 0.05 was considered statistically significant.

Patients from the same family have been included in the analysis, but may not be independent. To test the robustness of the findings, sensitivity analyses were conducted using a dataset restricted to one patient per family (the proband). Any discrepancies between the primary and sensitivity analyses are reported in the text.

### 3.4. Ethical Considerations

This study was approved by the Te Whatu Ora/Health New Zealand Wellington Hospital local research office, and was conducted in accordance with the ethical principles of the Declaration of Helsinki. Informed consent was obtained from the participant for publication of clinical and genetic data.

## 4. Results

### 4.1. WDR19 Cases

**Figure 2:**
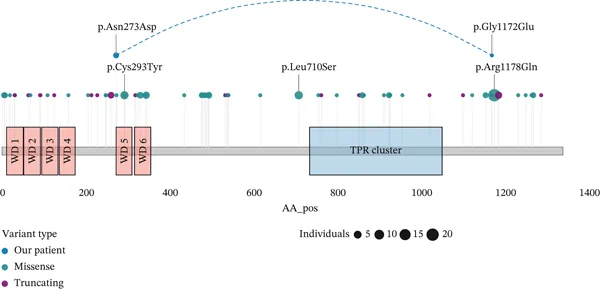
Distribution of reported *WDR19* variants, depicting WD repeat domains (1–6) and tetratricopeptide (TPR) repeat cluster.

Ninety‐three individuals from 83 families were identified from the literature; variant details including in silico predictions are displayed in Table S2. Figure 2 maps the location of the 84 unique variants.

Statistical analysis was restricted to 72 individuals from 64 families with biallelic *WDR19* variants, where at least one was classified as LP/P (Table 1). Of the 62 variants reported, 33/62 (53%) were missense, 17/62 truncating (27%; 10 frameshift, 7 nonsense) and 10/62 (16%) predicted to affect splicing. Additionally, there was 1 multiexon intragenic deletion and 1 in‐frame duplication.

**Table 1:** Biallelic *WDR19* variant combinations from 72 reported patients. Domain location was included for missense/missense and missense/truncating variants (*n* = 54); ‘repeat’ refers to location within repeat protein domains. Clinical manifestations are categorised into age of first manifestation (under or over 16 years) and organ‐specific involvement. For skeletal involvement, a separate analysis was done for both skeletal features of any type and thoracic dysplasia.

	*n*	%
**Variant combination**		
Missense/missense	30	42
Missense/truncating	24	33
Missense/other	18	25
**Domain combination**		
Repeat only	19	26
Repeat/not repeat	4	6
Not repeat only	31	43
Not applicable/available	18	25
**Age at first manifestation**		
≤ 16 years	57	79
> 16 years	9	13
Unclear/unknown from available clinical data	6	8
**Phenotype categories**		
Renal	54	75
Ocular	37	51
Skeletal—all	29	40
Skeletal—thorax	21	29
Hepatobiliary	28	39
Developmental/cognitive	14	19
Cardiac	5	7
Total	72	100

### 4.2. Genotype–Phenotype Correlations

To evaluate genotype–phenotype correlations, we examined the relationship between variant/domain combinations and phenotypic categories, including age of symptom onset.

#### 4.2.1. Type of Variant

**Figure 3:**
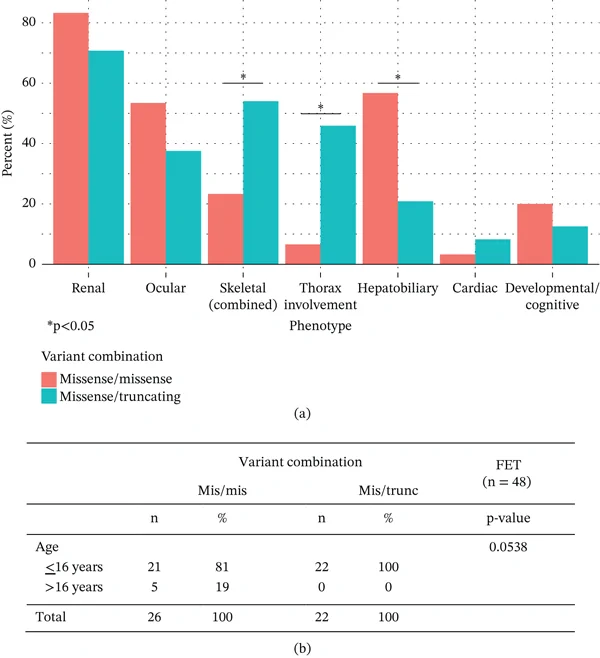
Relationship between variant type and phenotype. (a) Organ involvement and (b) age of onset. Data on age of onset was limited to 48 individuals; expected counts were small, so Fisher′s exact test (FET) was used. Mis, missense; trunc, truncating.

Individuals with a truncating variant were significantly more likely to have skeletal involvement than those with only missense variants, both overall (any skeletal involvement: 54% truncating vs. 23% missense only; *p* = 0.0191, OR = 3.88, 95% CI 1.24–13.09) and for thoracic involvement specifically (46% truncating vs 7% missense only; *p* = 0.0006, OR = 11.85, 95% CI 2.70–84.11; Figure 3a and Table S3). In contrast, individuals with a truncating variant were less likely to have hepatobiliary involvement compared with those with only missense variants (21% truncating vs. 57% missense only; *p* = 0.0066, OR = 0.20, 95% CI 0.05–0.65).

Sensitivity analyses restricted to one individual per family (the proband) yielded results consistent with the primary analysis. No significant associations were detected between variant type and other phenotypic categories, including age at presentation (Figure 3b).

#### 4.2.2. Location of Variant

Amongst patients with missense variants, domain location was significantly associated with age at symptom onset (*p* = 0.0042). Bonferroni‐adjusted pairwise comparisons determined that the presence of two variants located outside repeat domains was associated with an earlier age at symptom onset (100% not‐repeat‐only combinations were < 16 years, compared with 50% repeat/not repeat and 81% repeat‐only, both *p* < 0.05, Table S4). Similarly, ocular phenotypes were significantly associated with domain location (*p* < 0.0001); more frequent in individuals with at least one variant outside a repeat domain (repeat/not repeat 75%, not‐repeat‐only 68%, repeat‐only 5%).

The likelihood of hepatobiliary involvement also differed by domain location (*p* = 0.0215). There was a difference between the repeat/not repeat group (50%) versus repeat only group (11%), OR = 4.47, 95% CI 1.12–20.94; however, no significant difference was observed for not‐repeat‐only combinations.

## 5. Discussion

To our knowledge, this study provides the most comprehensive compilation and review of *WDR19* variants to date. We undertook analysis on genotype–phenotype correlations in over 90 cases diagnosed worldwide, including the 70 patients listed by Tang et al. [4]. The genotypic spectrum is broad, and although several recurrent variants have been reported, there are no clear regional hotspots. There have been no individuals or families reported with two truncating variants, supporting the theory that homozygous or compound heterozygous truncating variants may result in embryonic lethality [1]. Clinical data highlight the phenotypic variability of *WDR19*‐associated ciliopathies, with renal, retinal and skeletal manifestations most common (Table 1 and Table S1). Several explanations have been proposed for the range of presentations and severity associated with recurrent *WDR19* mutations, including oligogenic inheritance, modifier genes and environmental interactions [1, 7].

### 5.1. Recurrent Variants

The most common *WDR19* mutation is c.3533G > A (p.Arg1178Gln), reported in 26 cases from 24 families [3, 4, 9, 19–29]. Eight of these individuals were homozygous, of which 6/8 had end‐stage renal failure (ESRF) in early childhood and 4/6 had retinal dystrophy; the two homozygous cases published by Park 2015 did not include individualised clinical data on nonrenal phenotypes [9, 19, 20, 23, 24, 30].

The second most common mutations are c.3703G > A (p.Glu1235Lys) and c.817A > G (p.Asn273Asp), the latter identified in our patient and reported in six individuals from four families to date. All seven individuals with c.3703G > A had both renal and hepatic involvement [9, 19, 20, 27, 29]. c.817A > G has been reported in a compound heterozygous state with nonsense variant c.275 T > G (p.Leu92 ^∗^) in an infant with congenital absence of the left kidney and NPHP [31], frameshift variant c.1630_1639del (p.Val544Leufs ^∗^72) in an individual with Jeune syndrome [32], frameshift variant c.749C > G (p.Ser250 ^∗^) in a case of short rib thoracic dysplasia Type 5 [33], splice‐site variant c.3565+1G > A (p.?) in Jeune syndrome and missense c.3800G > A (p.Cys1267Tyr) in Jeune syndrome [34].

The splice‐site variant c.3565+1G > A is reported in five individuals from four families all in a compound heterozygous state: with c.14 T > C (p.Phe5Ser) in identical twins with SLS, and in an individual with ESRF secondary to NPHP (no other organ involvement reported) [28, 30]. Halbritter et al. reported the variant in combination with c.3533G > A (p.Arg1178Gln) in a patient with infantile‐onset ESRF, pancreatic cysts and cortical blindness [19].

The missense variant c.878G > A (p.Cys293Tyr) is reported in a homozygous state in five unrelated individuals of an Israeli ethnic minority group with adult‐onset ESRF [35]. Prior to molecular testing, most patients in their cohort had a clinical diagnosis of chronic kidney disease secondary to ‘unknown aetiology’. Aside from one individual with hepatic cirrhosis and portal hypertension, none of those cases were reported to have extrarenal manifestations. The authors concluded the variant causes a nonsyndromic kidney‐predominant phenotype.

### 5.2. WDR19 Clinical Features

#### 5.2.1. Renal

Renal manifestations were the most common phenotype associated with *WDR19* variants in our study, amongst all reported cases and those included in the subanalysis. Consistent with previous reports, nephronophthisis‐like nephropathy and/or cystic kidney disease were the most consistent features [4, 9, 22]. The spectrum of severity ranged from ESRF in infancy to mild proteinuria detected incidentally in adulthood [19, 22, 36]. Progression to ESRF was documented in a significant proportion of the cohort (44/94 individuals), implying that renal complications are a major cause of morbidity associated with *WDR19* variants.

Our patient presented with bland urinalysis, normal‐sized kidneys and gradual decline in renal function, and had been diagnosed with IgA nephropathy prior to molecular testing. Histology patterns reported with *WDR19* include interstitial fibrosis, atrophic tubules, basement membrane disintegration and glomerulosclerosis. Immune complex deposits have been reported in a small number of cases, with potential to mimic an immune‐complex mediated glomerulonephropathy [9, 35, 36].

Statistical analysis of 72 cases did not identify genetic features predictive of renal disease onset.

#### 5.2.2. Skeletal

A total of 36% of individuals in the total cohort had a skeletal phenotype, of which 79% had thoracic involvement. Bredrup et al. were able to demonstrate dysfunctional ciliogenesis and IFT protein localisation in the cilia of fibroblasts from a Sensenbrenner patient, validating ciliary dysfunction [1]. They proposed that a combination of two mutations severely impacting protein function may result in a more complex or severe phenotype, such as the perinatal lethal short‐rib polydactyly syndromes (SRPS). In support of this, our subanalysis demonstrated that individuals with a truncating variant were more likely to have skeletal involvement and specifically more likely to have a thoracic dysplasia than those with missense/missense combinations (Figure 3).

Of the total 94 *WDR19* cases identified, 9/94 were diagnosed with Sensenbrenner syndrome, 8/94 with Jeune, 3/94 with short‐rib thoracic dysplasia Type 5 (SRTD5), 1/94 with short‐rib polydactyly syndrome Type 4 (SRPS4) and 2 with a thoracic dysplasia not otherwise specified (Table S1) [1, 21, 24, 25, 31–33, 36–41]. Two cases of Mainzer–Saldino syndrome (MSS/MZSDS) have been reported in association with *WDR19* variants [3, 42].

#### 5.2.3. Eye

A total of 42% of the total cohort and 51% of individuals in the subanalysis had an ocular phenotype, the second most common organ involvement after kidneys. Coussa et al. originally described seven *WDR19* mutations in five families with SLS [7]. They noted a distinct ‘bear claw’ maculopathy seen on fundus autofluorescence in two separate French‐Canadian families with retinitis pigmentosa and hypothesised a novel genotype–phenotype correlation with homozygous missense mutation c.2129 T > C (p.Leu710Ser). Subsequent cases of SLS described in association with *WDR19* have not reported this variant nor a ‘bear claw’ pattern of maculopathy [26, 28, 43].

Our patient has concurrent nephropathy and retinal dystrophy; however, she lacks the widespread peripheral retinal changes reported in *WDR19-*associated SLS. Her ocular findings could be considered a variant of Stargardt disease: A condition characterised by progressive degeneration of the retinal pigment epithelium and photoreceptors, most often caused by biallelic pathogenic variants in *ABCA4* [44]. Several publications have reported a Stargardt‐like phenotype associated with *WDR19*, both in adult‐onset nonsyndromic form and in the presence of multisystemic disease [10, 28, 44–46].

Notably, ocular involvement was more common amongst individuals with variants outside repeat domains. This finding is unexplained, given the wide‐ranging functional roles of WD repeat proteins [47, 48]. Broadly speaking, pathogenic missense variants are more likely to be disruptive if localised within core protein structures and are more commonly enriched within intradomain ordered regions [49]. Our unexpected observation should be interpreted cautiously and may reflect limitations of domain annotation or underlying cohort characteristics rather than a true biological effect. Further functional studies are required to infer causation.

#### 5.2.4. Liver

Hepatobiliary involvement in *WDR19*‐related ciliopathies ranges from mildly elevated liver enzymes and hepatosplenomegaly to hepatic cirrhosis. All cases with a liver phenotype reported had concurrent renal involvement. The presence of both renal and liver involvement in ciliopathies has been referred to as ‘hepatorenal fibrocystic diseases’ (HRFCDs), with pathology further categorised into congenital hepatic fibrosis, Caroli disease and polycystic liver disease [9, 20]. Caroli disease refers to focal dilatation of the intrahepatic bile ducts and may be referred to as Caroli syndrome when congenital hepatic fibrosis is present [20, 50].

Lee et al. reported a cohort of six Korean individuals with NPHP‐13 and Caroli disease [9]. They speculated that Caroli disease may be a major extrarenal phenotype associated with *WDR19,* as 13/27 cases of NPHP‐13 published at the time reported accompanying liver involvement. An additional 14 cases of bile duct dilatation (including our patient) have since been reported in association with *WDR19* [4, 22, 23, 25, 26, 30, 37, 42, 50, 51]. Lee and colleagues further proposed a possible correlation between the recurrent missense variant c.3533G > A (p.Arg1178Gln) and liver phenotypes. Our study supports the high prevalence of liver involvement with c.3533G > A, in 23/36 (88%) of individuals with this variant compared with 31% of the total cohort and 29% of the subanalysis. However, our results indicate that hepatobiliary involvement is associated with missense variants more broadly. Even after exclusion of the recurrent c.3533G > A variant, individuals with missense‐only combinations remained significantly more likely than those with truncating variants to have hepatobiliary involvement (*p* = 0.0381, Table S5).

### 5.3. Study Limitations

There are inherent limitations in interpreting data from multiple sources. Details on clinical phenotype may be incomplete for some cases as it relies on individual clinician/authors′ reporting, with variation in segregation data and length of follow‐up. The potential for multisystem involvement in ciliopathies contributes to this challenge, as some clinical manifestations may be absent at the time of initial diagnosis. The relatively small size of patient cohorts for both rare and novel variants also poses challenges for statistical analysis, potentially reducing overall statistical power and resulting in wide confidence intervals. In addition, the statistical analyses include multiple individuals from the same family, likely violating the assumption of independent data. Additional sensitivity analysis was undertaken to assess whether including multiple relatives versus the proband alone affected the results.

Inaccurate determination of variants′ pathogenicity is another potential source of error when assessing genotype‐phenotype correlations. This strategy may have introduced both underascertainment and overascertainment. Some excluded individuals may represent true cases with undetected pathogenic variants, whereas some included LP/P + VUS cases may not be genuinely affected if the VUS is ultimately benign. This limitation reflects a necessary balance between sensitivity and specificity in the setting of incomplete and evolving variant classification.

Genetic analysis in our patient utilised a targeted NGS panel limited to genes associated with young‐onset kidney disease. This panel did not include all known ciliopathy‐associated genes described in the current literature; broader testing such as a whole‐exome or whole‐genome approach would be beneficial to exclude alternative genetic diagnoses. The proband′s affected sibling was unavailable for segregation of the variants and further clinical assessment, which limits further interpretation of phenotypic variability within the family.

## 6. Conclusion

In summary, we report a 32‐year‐old woman with nephropathy, retinal dystrophy and Caroli disease attributed to compound heterozygous *WDR19* variants, and review the clinical and molecular data from 93 previously published individuals. Our study reinforces the heterogeneity of *WDR19*‐related ciliopathies, with various combinations of missense, truncating and splice‐site variants reported and distributed throughout the gene, in addition to a small number of indels and copy number variants. Clinical presentations range from neonatal multisystemic disease to adult‐onset single organ involvement, emphasising the utility of molecular testing to aid diagnosis. We further examined variant combinations and clinical features from 72 cases where at least one *WDR19* variant has been classified as LP/P. Our analysis indicates a potential association between missense/truncating variant combinations and skeletal phenotypes, and missense/missense combinations with hepatobiliary involvement. Variants outside repeat protein domains correlated with ocular phenotypes and paediatric onset, although the small sample size is a limitation. Prognostication in *WDR19*‐associated ciliopathies remains challenging, with implications for care including awareness of organ systems that would benefit from early screening (e.g., kidney, eyes and liver), and genetic counselling for affected individuals to inform reproductive planning.

## Author Contributions

Z.W.: study design, data collection and writing (main author). G.P.: study design, supervision and writing (editing/review of manuscript). L.W.: statistical analysis and writing (editing/review of manuscript). K.D.: variant curation for selected cases and writing (editing/review of manuscript). R.D.: variant curation for selected cases. G.V‐l.: variant map.

## Funding

No funding was received for this manuscript.

## Conflicts of Interest

The authors declare no conflicts of interest.

## Supporting Information

Additional supporting information can be found online in the Supporting Information section.

## Supporting information


**Supporting Information 1** Table S1: Clinical data from the total cohort of 94 individuals. Patients included in the subanalysis are highlighted in bold.

**Supporting Information 2** Table S2: Variant data from the total cohort of 94 individuals. Patients included in the subanalysis are highlighted in bold.

**Supporting Information 3** Table S3: Statistical analysis of variant combinations (type of variant) and phenotype categories. Table S4: Statistical analysis of variant combinations (location) and phenotype categories. Table S5: Repeat statistical analysis of variant combinations (type of variant) and hepatobiliary involvement, with recurrent variant *WDR19*:c.3533G > A (p.Arg1178Gln) excluded.

## Data Availability

The data that supports the findings of this study are available in the Supporting Information of this article.
