# Supplementary material for: Biallelic WDR19 Variants: Systematic Analysis of Genotype–Phenotype Correlations
Source: Hum Mutat. 2026 Sep 17;2026:9449547. doi: 10.1155/humu/9449547 (PMC13583927; doi:10.1155/humu/9449547)
Supplement: Supplementary file 1 [file HUMU-2026-9449547-s001.docx]

**Supplementary Table S3: relationship between variant type combinations and phenotype categories,** for 54/72 individuals with either missense/missense or missense/truncating variant combinations.

| Phenotype category/organ system involved | Variant Combination | | | | Results (n=54) | | | |
| --- | --- | --- | --- | --- | --- | --- | --- | --- |
|  | mis/mis | | mis/trunc | | OR (95% CI)  mis/trunc vs mis/mis | Likelihood Ratio Test | |  |
|  | n | % | n | % |  | *X^2^*(1) | p-value |  |
| Renal | 25 | 83 | 17 | 71 | 0.49  (0.12, 1.77) | 1.20 | 0.2733 |  |
| Ocular | 16 | 53 | 9 | 38 | 0.53  (0.17, 1.55) | 1.35 | 0.2448 |  |
| Skeletal – combined | 7 | 23 | 13 | 54 | 3.88  (1.24, 13.09) | 5.49 | **0.0191*** |  |
| Thorax involvement | 2 | 7 | 11 | 46 | 11.85  (2.70, 84.11) | 11.81 | **0.0006*** |  |
| Hepatobiliary | 17 | 57 | 5 | 21 | 0.20  (0.05, 0.65) | 7.38 | **0.0066*** |  |
| Cardiac | 1 | 3 | 2 | 8 | 2.64  (0.24, 58.89) | 0.64 | 0.4253 |  |
| Developmental/ Cognitive | 6 | 20 | 3 | 13 | 0.57  (0.11, 2.45) | 0.55 | 0.4577 |  |
| Total | 30 | 100 | 24 | 100 |  |  |  |  |

OR = Odds Ratio; CI = Confidence Interval; mis: missense variant; trunc: truncating variant. *p<0.05

**Supplementary Table S4:** comparison between location of variant combinations within or outside repeat domains against phenotype categories. **(a)** organ involvement **(b)** age of onset. Bonferroni-adjusted pairwise comparisons determined that not repeat only variant combinations were significantly more likely to have congenital features or manifestations prior to age 16 years than repeat/not repeat combinations.

| Phenotype categories | Domain | | | | | | Results (n=54) | | | | |
| --- | --- | --- | --- | --- | --- | --- | --- | --- | --- | --- | --- |
|  | Repeat only | | Repeat/not repeat | | Not repeat only | | OR (95% CI) | | | LRT | |
|  | n | % | n | % | n | % | Repeat/not repeat vs Repeat only | Not repeat only vs repeat only | Not repeat only versus Repeat/Not repeat | *X^2^*(2) | p-value |
| Renal | 17 | 90 | 2 | 50 | 23 | 74 | 0.12  (0.01, 1.40) | 0.34  (0.05, 1.56) | 2.87  (0.30, 27.45) | 3.47 | 0.1761 |
| Ocular | 1 | 5 | 3 | 75 | 21 | 68 | 54.00  (3.69, 2268.86) | 37.80  (6.42, 729.77) | 0.70  (0.03, 6.29) | 23.24 | **<0.0001*** |
| Skeletal – combined | 6 | 32 | 2 | 50 | 12 | 39 | 2.17  (0.22, 21.98) | 1.37  (0.42, 4.79) | 0.63  (0.07, 5.84) | 0.56 | 0.7544 |
| Thorax involvement | 5 | 26 | 1 | 25 | 7 | 23 | 0.93  (0.04, 9.49) | 0.82  (0.22, 3.22) | 0.87  (0.09, 19.24) | 0.09 | 0.9553 |
| Hepatobiliary | 2 | 11 | 2 | 50 | 18 | 58 | 4.47  (1.12, 20.94) | 0.61  (0.07, 3.92) | 1.38  (0.15, 12.80) | 7.68 | **0.0215*** |
| Cardiac | 2 | 11 | 1 | 25 | 0 | 0 | 3.00  (0.22, 31.45) | 0.11  (0.00, 1.47) | 0.04  (0.00, 0.83) | 5.16 | 0.0760 |
| Developmental/ Cognitive | 1 | 5 | 0 | 0 | 8 | 26 | 1.37  (0.01, 30.72) | 4.46  (0.88, 44.62) | 3.26  (0.29, 448.63) | 3.51 | 0.1732 |
| Total | 19 | 100 | 4 | 100 | 31 | 100 |  |  |  |  |  |

(a)

OR = Odds Ratio; CI = Confidence Interval; LRT = Likelihood Ratio Test. + Due to separation in the data, the logistic regression model uses Firth's bias reduction method. * p < 0.05

(b)

|  | **Domain (missense variants only)** | | | |  |  | FET |
| --- | --- | --- | --- | --- | --- | --- | --- |
|  | Repeat only | | Repeat/not repeat | | Not repeat only | |  |
|  | n | % | n | % | n | % | p-value |
| Age  ≤16 years  >16 years | 13  3 | 81  19 | 2  2 | 50  50 | 28  0 | 100  0 | **0.0042*** |
| Total | 16 | 100 | 4 | 100 | 28 | 100 |  |

* p < 0.05

**Supplementary Table S5: relationship between variant type combinations and hepatobiliary phenotype;** repeat analysis with individuals with recurrent variant *WDR19*:c.3533G>A excluded

| Phenotype category/organ system involved | Variant Combination | | | | Results (n=37) | | | |
| --- | --- | --- | --- | --- | --- | --- | --- | --- |
|  | Mis/mis | | Mis/trunc | | OR (95% CI)  B vs A | Likelihood Ratio Test | |  |
|  | n | % | n | % |  | *X^2^*(1) | p-value |  |
| Hepatobiliary | 6 | 38 | 2 | 10 | 0.18  (0.02, 0.92) | 4.26 | **0.0391*** |  |
| Total | 16 | 100 | 21 | 100 |  |  |  |  |

OR = Odds Ratio; CI = Confidence Interval, mis: missense variant, trunc: truncating variant, * p < 0.05
